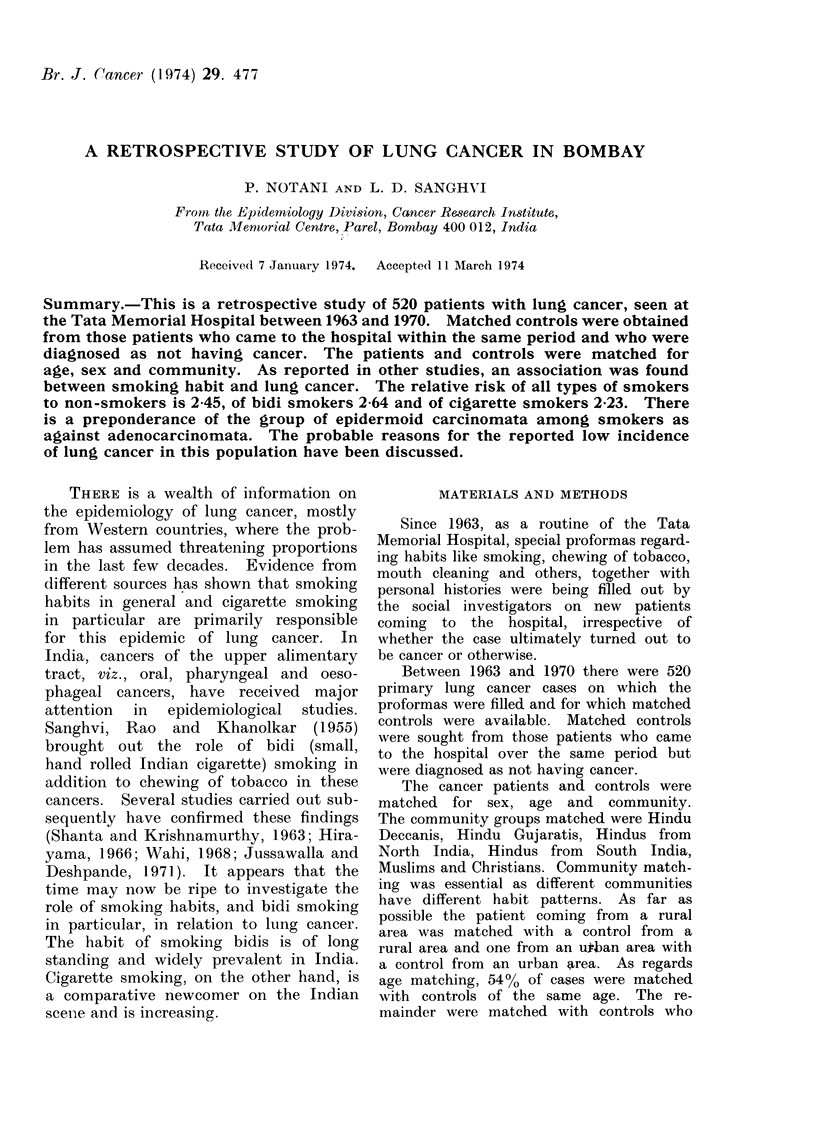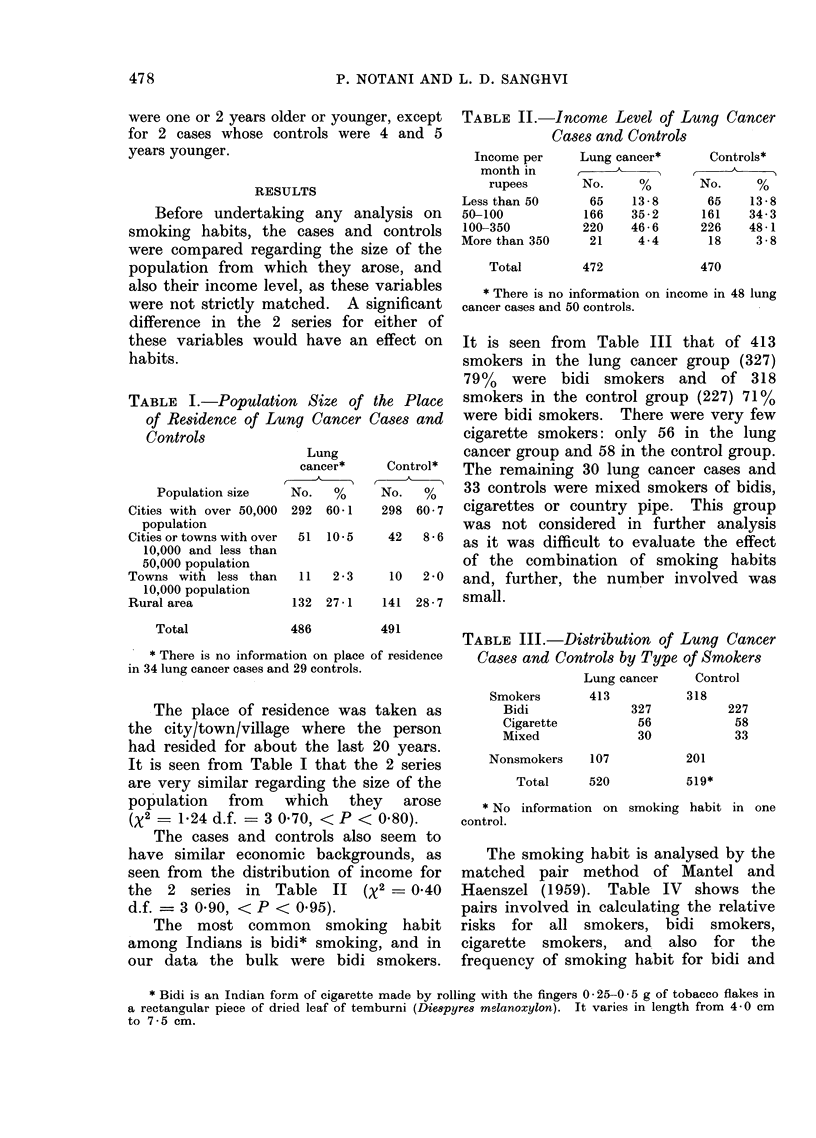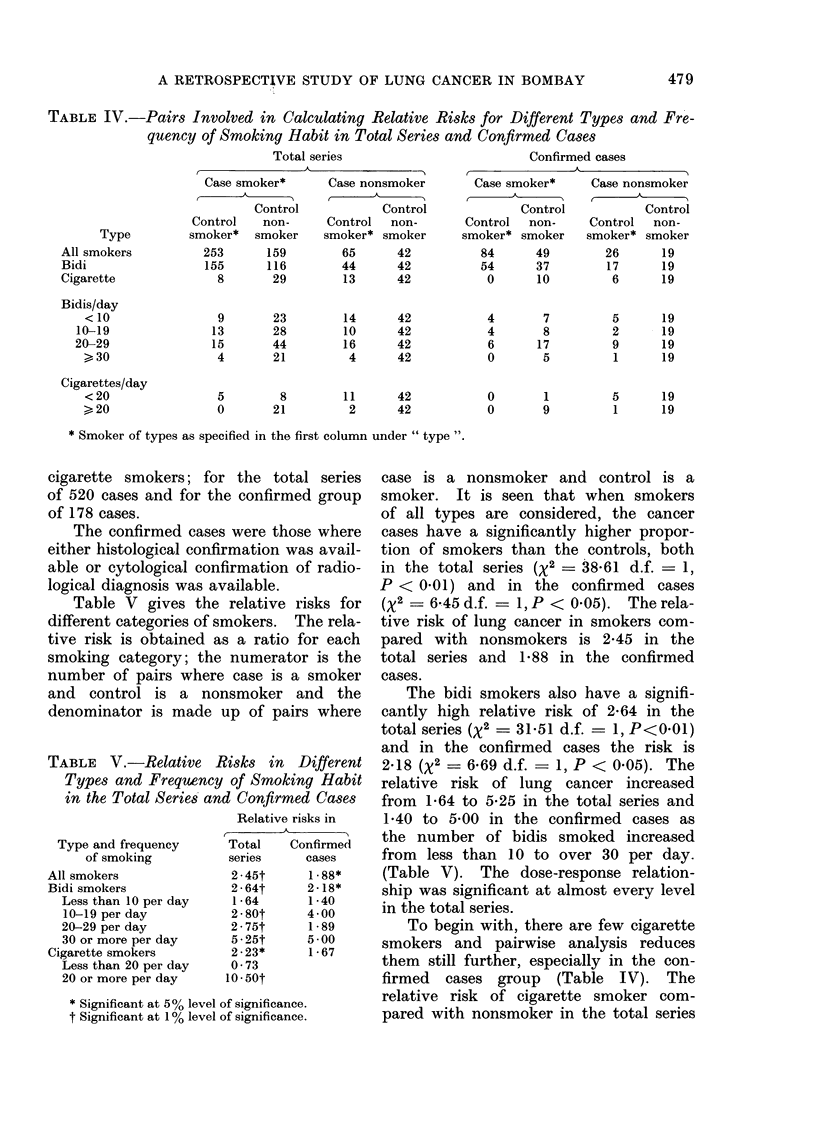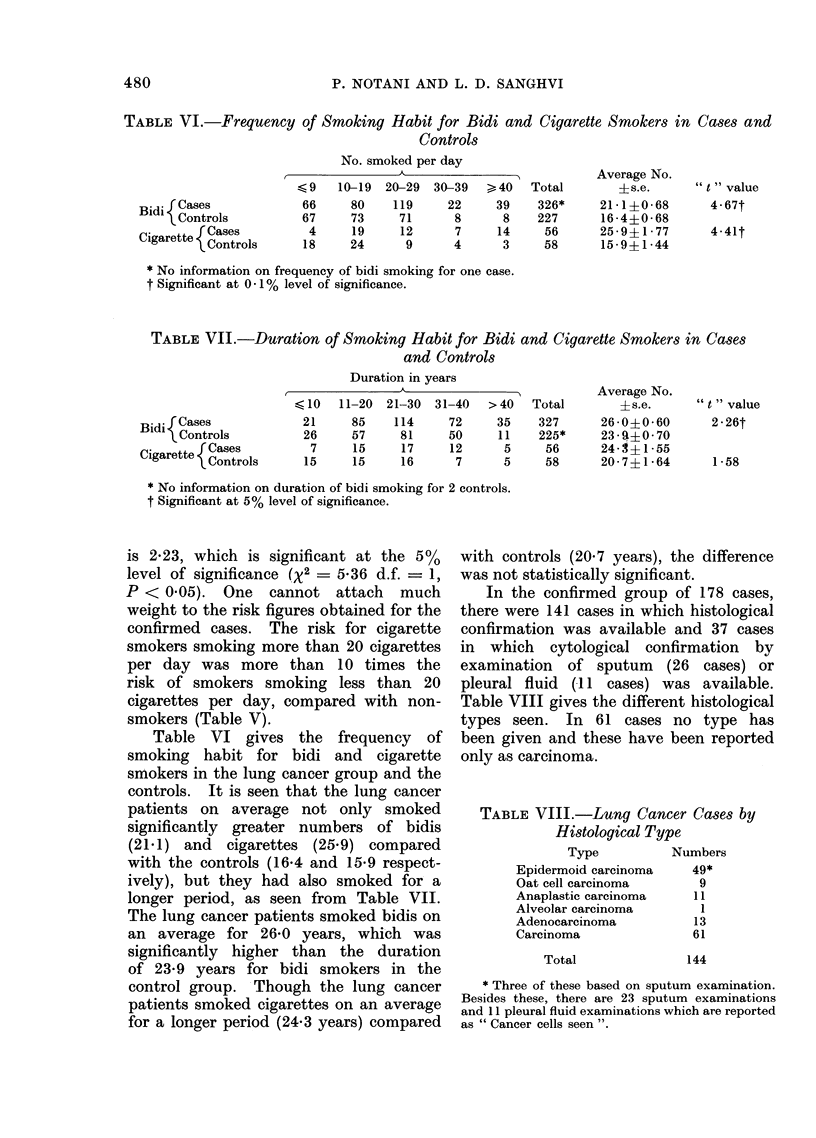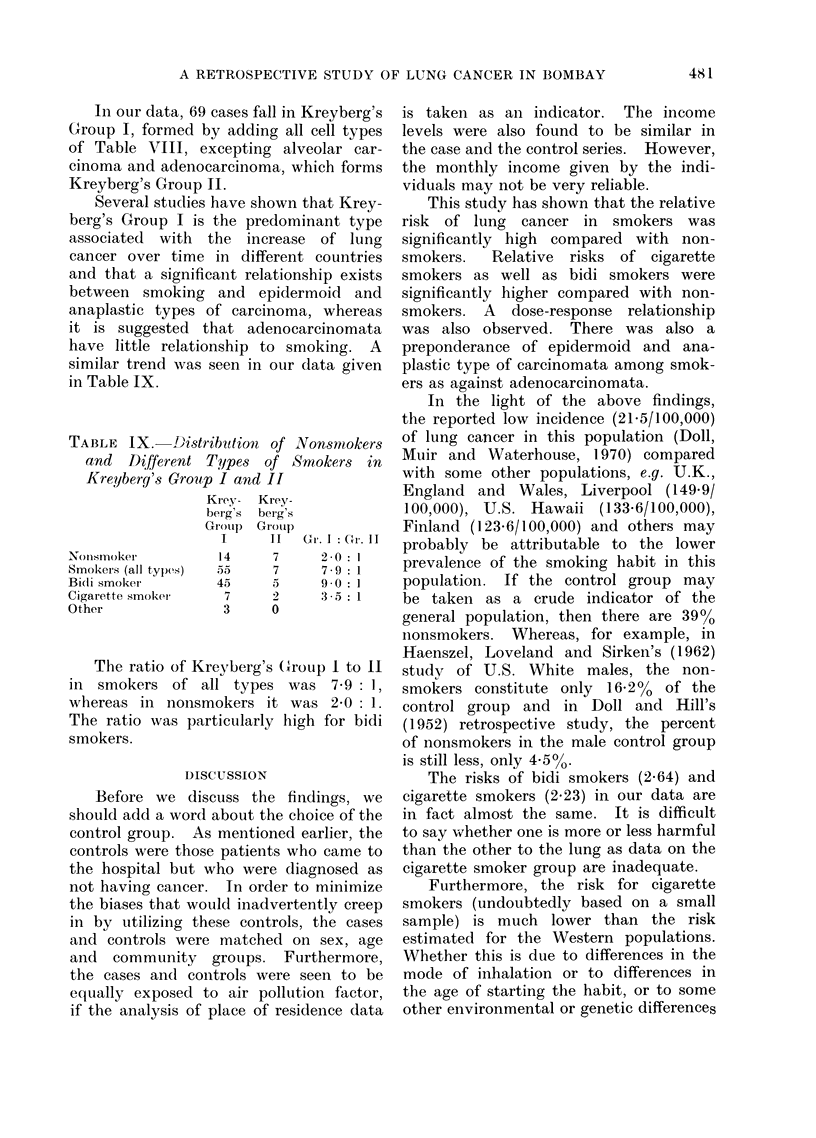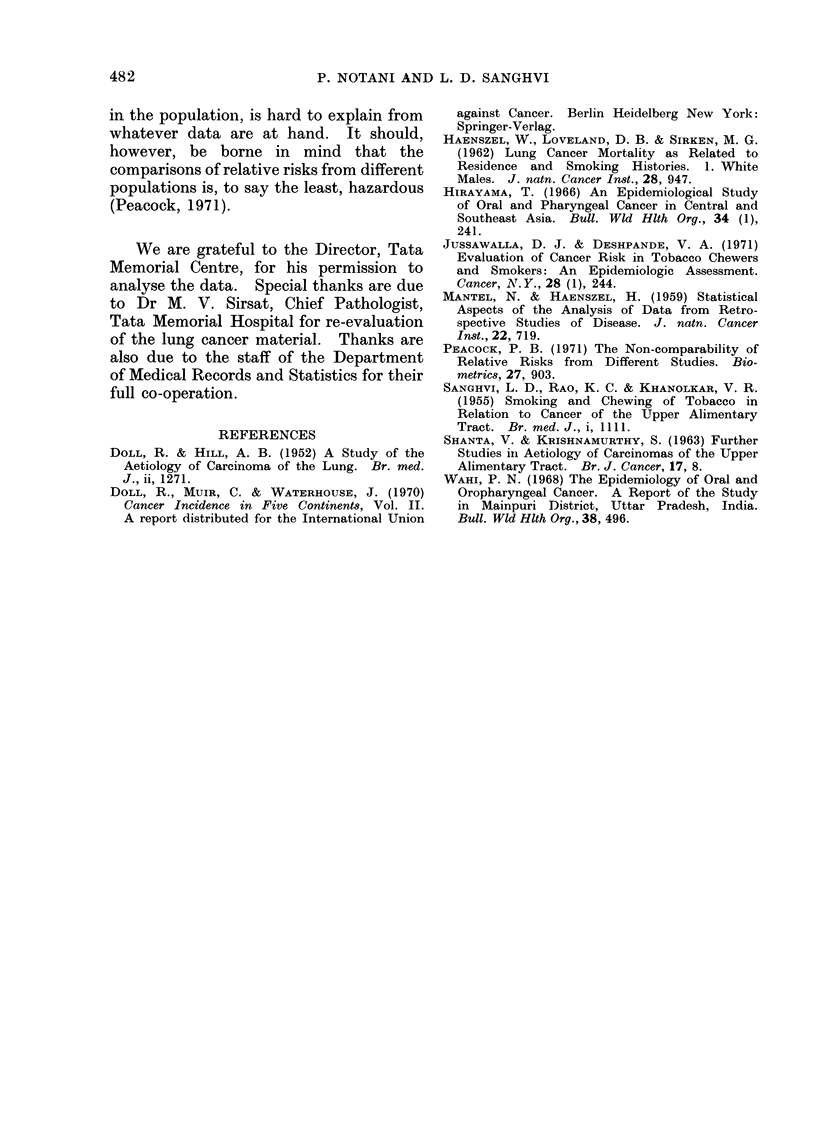# A Retrospective Study of Lung Cancer in Bombay

**DOI:** 10.1038/bjc.1974.100

**Published:** 1974-06

**Authors:** P. Notani, L. D. Sanghvi

## Abstract

This is a retrospective study of 520 patients with lung cancer, seen at the Tata Memorial Hospital between 1963 and 1970. Matched controls were obtained from those patients who came to the hospital within the same period and who were diagnosed as not having cancer. The patients and controls were matched for age, sex and community. As reported in other studies, an association was found between smoking habit and lung cancer. The relative risk of all types of smokers to non-smokers is 2·45, of bidi smokers 2·64 and of cigarette smokers 2·23. There is a preponderance of the group of epidermoid carcinomata among smokers as against adenocarcinomata. The probable reasons for the reported low incidence of lung cancer in this population have been discussed.


Br. J. Cancer (1974) 29. 477

### A RETROSPECTIVE STUDY OF LUNG CANCER IN BOMBAY

P. NOTANI AND L. D. SANGHVI

*From the Epidemiology Division, Cancer Research Institute, Tata Memorial Centre, Parel, Bombay 400 012, India*

Received 7 January 1974. Accepted 11 March 1974

**Summary.—This is a retrospective study of 520 patients with lung cancer, seen at the Tata Memorial Hospital between 1963 and 1970. Matched controls were obtained from those patients who came to the hospital within the same period and who were diagnosed as not having cancer. The patients and controls were matched for age, sex and community. As reported in other studies, an association was found between smoking habit and lung cancer. The relative risk of all types of smokers to non-smokers is 2·45, of bidi smokers 2·64 and of cigarette smokers 2·23. There is a preponderance of the group of epidermoid carcinomata among smokers as against adenocarcinomata. The probable reasons for the reported low incidence of lung cancer in this population have been discussed.**

THERE is a wealth of information on the epidemiology of lung cancer, mostly from Western countries, where the problem has assumed threatening proportions in the last few decades. Evidence from different sources has shown that smoking habits in general and cigarette smoking in particular are primarily responsible for this epidemic of lung cancer. In India, cancers of the upper alimentary tract, *viz.*, oral, pharyngeal and oesophageal cancers, have received major attention in epidemiological studies. Sanghvi, Rao and Khanolkar (1955) brought out the role of bidi (small, hand rolled Indian cigarette) smoking in addition to chewing of tobacco in these cancers. Several studies carried out subsequently have confirmed these findings (Shanta and Krishnamurthy, 1963; Hirayama, 1966; Wahi, 1968; Jussawalla and Deshpande, 1971). It appears that the time may now be ripe to investigate the role of smoking habits, and bidi smoking in particular, in relation to lung cancer. The habit of smoking bidis is of long standing and widely prevalent in India. Cigarette smoking, on the other hand, is a comparative newcomer on the Indian scene and is increasing.

### MATERIALS AND METHODS

Since 1963, as a routine of the Tata Memorial Hospital, special proformas regarding habits like smoking, chewing of tobacco, mouth cleaning and others, together with personal histories were being filled out by the social investigators on new patients coming to the hospital, irrespective of whether the case ultimately turned out to be cancer or otherwise.

Between 1963 and 1970 there were 520 primary lung cancer cases on which the proformas were filled and for which matched controls were available. Matched controls were sought from those patients who came to the hospital over the same period but were diagnosed as not having cancer.

The cancer patients and controls were matched for sex, age and community. The community groups matched were Hindu Deccanis, Hindu Gujaratis, Hindus from North India, Hindus from South India, Muslims and Christians. Community matching was essential as different communities have different habit patterns. As far as possible the patient coming from a rural area was matched with a control from a rural area and one from an urban area with a control from an urban area. As regards age matching, 54% of cases were matched with controls of the same age. The remainder were matched with controls who were one or 2 years older or younger, except for 2 cases whose controls were 4 and 5 years younger.

### RESULTS

Before undertaking any analysis on smoking habits, the cases and controls were compared regarding the size of the population from which they arose, and also their income level, as these variables were not strictly matched. A significant difference in the 2 series for either of these variables would have an effect on habits.

TABLE I.—*Population Size of the Place of Residence of Lung Cancer Cases and Controls*

| Population size | Lung cancer* No. | Lung cancer* % | Control* No. | Control* % |
|---|---|---|---|---|
| Cities with over 50,000 population | 292 | 60·1 | 298 | 60·7 |
| Cities or towns with over 10,000 and less than 50,000 population | 51 | 10·5 | 42 | 8·6 |
| Towns with less than 10,000 population | 11 | 2·3 | 10 | 2·0 |
| Rural area | 132 | 27·1 | 141 | 28·7 |
| Total | 486 | | 491 | |

* There is no information on place of residence in 34 lung cancer cases and 29 controls.

The place of residence was taken as the city/town/village where the person had resided for about the last 20 years. It is seen from Table I that the 2 series are very similar regarding the size of the population from which they arose ($\chi^2 = 1{\cdot}24$ d.f. $= 3$ $0{\cdot}70, < P < 0{\cdot}80$).

The cases and controls also seem to have similar economic backgrounds, as seen from the distribution of income for the 2 series in Table II ($\chi^2 = 0{\cdot}40$ d.f. $= 3$ $0{\cdot}90, < P < 0{\cdot}95$).

The most common smoking habit among Indians is bidi* smoking, and in our data the bulk were bidi smokers.

TABLE II.—*Income Level of Lung Cancer Cases and Controls*

| Income per month in rupees | Lung cancer* No. | Lung cancer* % | Controls* No. | Controls* % |
|---|---|---|---|---|
| Less than 50 | 65 | 13·8 | 65 | 13·8 |
| 50–100 | 166 | 35·2 | 161 | 34·3 |
| 100–350 | 220 | 46·6 | 226 | 48·1 |
| More than 350 | 21 | 4·4 | 18 | 3·8 |
| Total | 472 | | 470 | |

* There is no information on income in 48 lung cancer cases and 50 controls.

It is seen from Table III that of 413 smokers in the lung cancer group (327) 79% were bidi smokers and of 318 smokers in the control group (227) 71% were bidi smokers. There were very few cigarette smokers: only 56 in the lung cancer group and 58 in the control group. The remaining 30 lung cancer cases and 33 controls were mixed smokers of bidis, cigarettes or country pipe. This group was not considered in further analysis as it was difficult to evaluate the effect of the combination of smoking habits and, further, the number involved was small.

TABLE III.—*Distribution of Lung Cancer Cases and Controls by Type of Smokers*

| | Lung cancer | | Control | |
|---|---|---|---|---|
| Smokers | 413 | | 318 | |
| Bidi | | 327 | | 227 |
| Cigarette | | 56 | | 58 |
| Mixed | | 30 | | 33 |
| Nonsmokers | 107 | | 201 | |
| Total | 520 | | 519* | |

* No information on smoking habit in one control.

The smoking habit is analysed by the matched pair method of Mantel and Haenszel (1959). Table IV shows the pairs involved in calculating the relative risks for all smokers, bidi smokers, cigarette smokers, and also for the frequency of smoking habit for bidi and

* Bidi is an Indian form of cigarette made by rolling with the fingers 0·25–0·5 g of tobacco flakes in a rectangular piece of dried leaf of temburni (*Diespyres melanoxylon*). It varies in length from 4·0 cm to 7·5 cm.

TABLE IV.—*Pairs Involved in Calculating Relative Risks for Different Types and Frequency of Smoking Habit in Total Series and Confirmed Cases*

| | Total series | | | | Confirmed cases | | | |
|---|---|---|---|---|---|---|---|---|
| | Case smoker* | | Case nonsmoker | | Case smoker* | | Case nonsmoker | |
| Type | Control smoker* | Control non-smoker | Control smoker* | Control non-smoker | Control smoker* | Control non-smoker | Control smoker* | Control non-smoker |
| All smokers | 253 | 159 | 65 | 42 | 84 | 49 | 26 | 19 |
| Bidi | 155 | 116 | 44 | 42 | 54 | 37 | 17 | 19 |
| Cigarette | 8 | 29 | 13 | 42 | 0 | 10 | 6 | 19 |
| Bidis/day | | | | | | | | |
| < 10 | 9 | 23 | 14 | 42 | 4 | 7 | 5 | 19 |
| 10–19 | 13 | 28 | 10 | 42 | 4 | 8 | 2 | 19 |
| 20–29 | 15 | 44 | 16 | 42 | 6 | 17 | 9 | 19 |
| ⩾30 | 4 | 21 | 4 | 42 | 0 | 5 | 1 | 19 |
| Cigarettes/day | | | | | | | | |
| < 20 | 5 | 8 | 11 | 42 | 0 | 1 | 5 | 19 |
| ⩾20 | 0 | 21 | 2 | 42 | 0 | 9 | 1 | 19 |

* Smoker of types as specified in the first column under " type ".

cigarette smokers; for the total series of 520 cases and for the confirmed group of 178 cases.

The confirmed cases were those where either histological confirmation was available or cytological confirmation of radiological diagnosis was available.

Table V gives the relative risks for different categories of smokers. The relative risk is obtained as a ratio for each smoking category; the numerator is the number of pairs where case is a smoker and control is a nonsmoker and the denominator is made up of pairs where case is a nonsmoker and control is a smoker. It is seen that when smokers of all types are considered, the cancer cases have a significantly higher proportion of smokers than the controls, both in the total series ($\chi^2 = 38{\cdot}61$ d.f. $= 1$, $P < 0{\cdot}01$) and in the confirmed cases ($\chi^2 = 6{\cdot}45$ d.f. $= 1, P < 0{\cdot}05$). The relative risk of lung cancer in smokers compared with nonsmokers is 2·45 in the total series and 1·88 in the confirmed cases.

TABLE V.—*Relative Risks in Different Types and Frequency of Smoking Habit in the Total Series and Confirmed Cases*

| | Relative risks in | |
|---|---|---|
| Type and frequency of smoking | Total series | Confirmed cases |
| All smokers | 2·45† | 1·88* |
| Bidi smokers | 2·64† | 2·18* |
| Less than 10 per day | 1·64 | 1·40 |
| 10–19 per day | 2·80† | 4·00 |
| 20–29 per day | 2·75† | 1·89 |
| 30 or more per day | 5·25† | 5·00 |
| Cigarette smokers | 2·23* | 1·67 |
| Less than 20 per day | 0·73 | |
| 20 or more per day | 10·50† | |

* Significant at 5% level of significance.
† Significant at 1% level of significance.

The bidi smokers also have a significantly high relative risk of 2·64 in the total series ($\chi^2 = 31{\cdot}51$ d.f. $= 1, P<0{\cdot}01$) and in the confirmed cases the risk is 2·18 ($\chi^2 = 6{\cdot}69$ d.f. $= 1$, $P < 0{\cdot}05$). The relative risk of lung cancer increased from 1·64 to 5·25 in the total series and 1·40 to 5·00 in the confirmed cases as the number of bidis smoked increased from less than 10 to over 30 per day. (Table V). The dose-response relationship was significant at almost every level in the total series.

To begin with, there are few cigarette smokers and pairwise analysis reduces them still further, especially in the confirmed cases group (Table IV). The relative risk of cigarette smoker compared with nonsmoker in the total series

TABLE VI.—*Frequency of Smoking Habit for Bidi and Cigarette Smokers in Cases and Controls*

| | | No. smoked per day | | | | | | | |
|---|---|---|---|---|---|---|---|---|---|
| | | ⩽9 | 10–19 | 20–29 | 30–39 | ⩾40 | Total | Average No. ±s.e. | " $t$ " value |
| Bidi | Cases | 66 | 80 | 119 | 22 | 39 | 326* | 21·1±0·68 | 4·67† |
| | Controls | 67 | 73 | 71 | 8 | 8 | 227 | 16·4±0·68 | |
| Cigarette | Cases | 4 | 19 | 12 | 7 | 14 | 56 | 25·9±1·77 | 4·41† |
| | Controls | 18 | 24 | 9 | 4 | 3 | 58 | 15·9±1·44 | |

* No information on frequency of bidi smoking for one case.
† Significant at 0·1% level of significance.

TABLE VII.—*Duration of Smoking Habit for Bidi and Cigarette Smokers in Cases and Controls*

| | | Duration in years | | | | | | | |
|---|---|---|---|---|---|---|---|---|---|
| | | ⩽10 | 11–20 | 21–30 | 31–40 | >40 | Total | Average No. ±s.e. | " $t$ " value |
| Bidi | Cases | 21 | 85 | 114 | 72 | 35 | 327 | 26·0±0·60 | 2·26† |
| | Controls | 26 | 57 | 81 | 50 | 11 | 225* | 23·9±0·70 | |
| Cigarette | Cases | 7 | 15 | 17 | 12 | 5 | 56 | 24·3±1·55 | |
| | Controls | 15 | 15 | 16 | 7 | 5 | 58 | 20·7±1·64 | 1·58 |

* No information on duration of bidi smoking for 2 controls.
† Significant at 5% level of significance.

is 2·23, which is significant at the 5% level of significance ($\chi^2 = 5{\cdot}36$ d.f. $= 1$, $P < 0{\cdot}05$). One cannot attach much weight to the risk figures obtained for the confirmed cases. The risk for cigarette smokers smoking more than 20 cigarettes per day was more than 10 times the risk of smokers smoking less than 20 cigarettes per day, compared with non-smokers (Table V).

Table VI gives the frequency of smoking habit for bidi and cigarette smokers in the lung cancer group and the controls. It is seen that the lung cancer patients on average not only smoked significantly greater numbers of bidis (21·1) and cigarettes (25·9) compared with the controls (16·4 and 15·9 respectively), but they had also smoked for a longer period, as seen from Table VII. The lung cancer patients smoked bidis on an average for 26·0 years, which was significantly higher than the duration of 23·9 years for bidi smokers in the control group. Though the lung cancer patients smoked cigarettes on an average for a longer period (24·3 years) compared with controls (20·7 years), the difference was not statistically significant.

In the confirmed group of 178 cases, there were 141 cases in which histological confirmation was available and 37 cases in which cytological confirmation by examination of sputum (26 cases) or pleural fluid (11 cases) was available. Table VIII gives the different histological types seen. In 61 cases no type has been given and these have been reported only as carcinoma.

TABLE VIII.—*Lung Cancer Cases by Histological Type*

| Type | Numbers |
|---|---|
| Epidermoid carcinoma | 49* |
| Oat cell carcinoma | 9 |
| Anaplastic carcinoma | 11 |
| Alveolar carcinoma | 1 |
| Adenocarcinoma | 13 |
| Carcinoma | 61 |
| Total | 144 |

* Three of these based on sputum examination. Besides these, there are 23 sputum examinations and 11 pleural fluid examinations which are reported as " Cancer cells seen ".

In our data, 69 cases fall in Kreyberg's Group I, formed by adding all cell types of Table VIII, excepting alveolar carcinoma and adenocarcinoma, which forms Kreyberg's Group II.

Several studies have shown that Kreyberg's Group I is the predominant type associated with the increase of lung cancer over time in different countries and that a significant relationship exists between smoking and epidermoid and anaplastic types of carcinoma, whereas it is suggested that adenocarcinomata have little relationship to smoking. A similar trend was seen in our data given in Table IX.

TABLE IX.—*Distribution of Nonsmokers and Different Types of Smokers in Kreyberg's Group I and II*

| | Kreyberg's Group I | Kreyberg's Group II | Gr. I : Gr. II |
|---|---|---|---|
| Nonsmoker | 14 | 7 | 2·0 : 1 |
| Smokers (all types) | 55 | 7 | 7·9 : 1 |
| Bidi smoker | 45 | 5 | 9·0 : 1 |
| Cigarette smoker | 7 | 2 | 3·5 : 1 |
| Other | 3 | 0 | |

The ratio of Kreyberg's Group I to II in smokers of all types was 7·9 : 1, whereas in nonsmokers it was 2·0 : 1. The ratio was particularly high for bidi smokers.

### DISCUSSION

Before we discuss the findings, we should add a word about the choice of the control group. As mentioned earlier, the controls were those patients who came to the hospital but who were diagnosed as not having cancer. In order to minimize the biases that would inadvertently creep in by utilizing these controls, the cases and controls were matched on sex, age and community groups. Furthermore, the cases and controls were seen to be equally exposed to air pollution factor, if the analysis of place of residence data is taken as an indicator. The income levels were also found to be similar in the case and the control series. However, the monthly income given by the individuals may not be very reliable.

This study has shown that the relative risk of lung cancer in smokers was significantly high compared with nonsmokers. Relative risks of cigarette smokers as well as bidi smokers were significantly higher compared with nonsmokers. A dose-response relationship was also observed. There was also a preponderance of epidermoid and anaplastic type of carcinomata among smokers as against adenocarcinomata.

In the light of the above findings, the reported low incidence (21·5/100,000) of lung cancer in this population (Doll, Muir and Waterhouse, 1970) compared with some other populations, *e.g.* U.K., England and Wales, Liverpool (149·9/100,000), U.S. Hawaii (133·6/100,000), Finland (123·6/100,000) and others may probably be attributable to the lower prevalence of the smoking habit in this population. If the control group may be taken as a crude indicator of the general population, then there are 39% nonsmokers. Whereas, for example, in Haenszel, Loveland and Sirken's (1962) study of U.S. White males, the nonsmokers constitute only 16·2% of the control group and in Doll and Hill's (1952) retrospective study, the percent of nonsmokers in the male control group is still less, only 4·5%.

The risks of bidi smokers (2·64) and cigarette smokers (2·23) in our data are in fact almost the same. It is difficult to say whether one is more or less harmful than the other to the lung as data on the cigarette smoker group are inadequate.

Furthermore, the risk for cigarette smokers (undoubtedly based on a small sample) is much lower than the risk estimated for the Western populations. Whether this is due to differences in the mode of inhalation or to differences in the age of starting the habit, or to some other environmental or genetic differences in the population, is hard to explain from whatever data are at hand. It should, however, be borne in mind that the comparisons of relative risks from different populations is, to say the least, hazardous (Peacock, 1971).

We are grateful to the Director, Tata Memorial Centre, for his permission to analyse the data. Special thanks are due to Dr M. V. Sirsat, Chief Pathologist, Tata Memorial Hospital for re-evaluation of the lung cancer material. Thanks are also due to the staff of the Department of Medical Records and Statistics for their full co-operation.

### REFERENCES

DOLL, R. & HILL, A. B. (1952) A Study of the Aetiology of Carcinoma of the Lung. *Br. med. J.*, ii, 1271.

DOLL, R., MUIR, C. & WATERHOUSE, J. (1970) *Cancer Incidence in Five Continents*, Vol. II. A report distributed for the International Union against Cancer. Berlin Heidelberg New York: Springer-Verlag.

HAENSZEL, W., LOVELAND, D. B. & SIRKEN, M. G. (1962) Lung Cancer Mortality as Related to Residence and Smoking Histories. 1. White Males. *J. natn. Cancer Inst.*, **28**, 947.

HIRAYAMA, T. (1966) An Epidemiological Study of Oral and Pharyngeal Cancer in Central and Southeast Asia. *Bull. Wld Hlth Org.*, **34** (1), 241.

JUSSAWALLA, D. J. & DESHPANDE, V. A. (1971) Evaluation of Cancer Risk in Tobacco Chewers and Smokers: An Epidemiologic Assessment. *Cancer, N.Y.*, **28** (1), 244.

MANTEL, N. & HAENSZEL, H. (1959) Statistical Aspects of the Analysis of Data from Retrospective Studies of Disease. *J. natn. Cancer Inst.*, **22**, 719.

PEACOCK, P. B. (1971) The Non-comparability of Relative Risks from Different Studies. *Biometrics*, **27**, 903.

SANGHVI, L. D., RAO, K. C. & KHANOLKAR, V. R. (1955) Smoking and Chewing of Tobacco in Relation to Cancer of the Upper Alimentary Tract. *Br. med. J.*, i, 1111.

SHANTA, V. & KRISHNAMURTHY, S. (1963) Further Studies in Aetiology of Carcinomas of the Upper Alimentary Tract. *Br. J. Cancer*, **17**, 8.

WAHI, P. N. (1968) The Epidemiology of Oral and Oropharyngeal Cancer. A Report of the Study in Mainpuri District, Uttar Pradesh, India. *Bull. Wld Hlth Org.*, **38**, 496.